# Carbon Transfer from the Host to *Tuber melanosporum* Mycorrhizas and Ascocarps Followed Using a ^13^C Pulse-Labeling Technique

**DOI:** 10.1371/journal.pone.0064626

**Published:** 2013-05-31

**Authors:** François Le Tacon, Bernd Zeller, Caroline Plain, Christian Hossann, Claude Bréchet, Christophe Robin

**Affiliations:** 1 INRA, UMR 1136, Interactions Arbres/Microorganismes (IAM), Centre INRA de Nancy, Champenoux, France; 2 Université de Lorraine, UMR 1136, Interactions Arbres/Microorganismes (IAM), Faculté des Sciences, Vandoeuvre les Nancy, France; 3 INRA, UR 1138, Biogéochimie des Ecosystèmes Forestiers (BEF), Centre INRA de Nancy, Champenoux, France; 4 INRA, UMR 1137, Ecologie et Ecophysiologie Forestières (EEF), Centre INRA de Nancy, Champenoux, France; 5 Université de Lorraine, UMR 1137, Ecologie et Ecophysiologie Forestières (EEF), Faculté des Sciences, Vandoeuvre les Nancy, France; 6 Université de Lorraine, UMR 1121 « Agronomie & Environnement » Nancy-Colmar, Vandœuvre les Nancy, France; 7 INRA, UMR 1121 « Agronomie & Environnement » Nancy-Colmar, Centre INRA de Nancy, Vandœuvre les Nancy, France; Roehampton University, United Kingdom

## Abstract

Truffles ascocarps need carbon to grow, but it is not known whether this carbon comes directly from the tree (heterotrophy) or from soil organic matter (saprotrophy). The objective of this work was to investigate the heterotrophic side of the ascocarp nutrition by assessing the allocation of carbon by the host to *Tuber melanosporum* mycorrhizas and ascocarps. In 2010, a single hazel tree selected for its high truffle (*Tuber melanosporum*) production and situated in the west part of the Vosges, France, was labeled with ^13^CO_2_. The transfer of ^13^C from the leaves to the fine roots and *T. melanosporum* mycorrhizas was very slow compared with the results found in the literature for herbaceous plants or other tree species. The fine roots primarily acted as a carbon conduit; they accumulated little ^13^C and transferred it slowly to the mycorrhizas. The mycorrhizas first formed a carbon sink and accumulated ^13^C prior to ascocarp development. Then, the mycorrhizas transferred ^13^C to the ascocarps to provide constitutive carbon (1.7 mg of ^13^C per day). The ascocarps accumulated host carbon until reaching complete maturity, 200 days after the first labeling and 150 days after the second labeling event. This role of the *Tuber* ascocarps as a carbon sink occurred several months after the end of carbon assimilation by the host and at low temperature. This finding suggests that carbon allocated to the ascocarps during winter was provided by reserve compounds stored in the wood and hydrolyzed during a period of frost. Almost all of the constitutive carbon allocated to the truffles (1% of the total carbon assimilated by the tree during the growing season) came from the host.

## Introduction

Despite their renown, the life cycle of the true truffles belonging to the genus *Tuber,* which are members of the Ascomycota, is not well understood. These species form ectomycorrhizas with different hosts [1], [2]. For sexual reproduction, it is hypothesized that haploid mycorrhizas of one mating type form an antheridium producing male gametes and, through an ascogonial filament or cord, an ascogonial apparatus composed of ascogonial cells and a trichogyne. The female haploid trichogyne of one mating type is assumed to collect the male gametes of the opposite mating type, allowing the ascogonial apparatus to form, after plasmogamy, an ascogenous heterokaryotic tissue, which appears to be surrounded by homokaryotic maternal tissues. The growth of these tissues gives rise to the ascocarp.

In contrast to ectomycorrhizal basidiomycota sporocarps, such as those of *Boletus*, *Amanita* or *Laccaria*, which develop over a number of days directly from diploid mycorrhizas [3], *Tuber* ascocarps grow more slowly [4].

It takes at least six months between the production of the primordia and full ascocarp development. We thus hypothesize that the processes involved in ascocarp development and carbon acquisition are different from those of basidiocarps. It is not known whether the developing ascocarp is fed *via* a direct transfer of carbohydrates from the host tree through the mycorrhizas and the ascogonial structure or whether the ascocarp becomes independent of its host some weeks or months after its development. In the latter case, it is assumed that truffles might be able to use dead host tissues or soil organic matter as carbon (C) and nitrogen (N) sources, as indicated by some authors [5], [6], through a saprotrophic process. This feeding behavior cannot be excluded because the truffle ascocarp can develop an external mycelium from its peridium. This mycelium could colonize dead cells from living roots, dead roots, other dead organic tissues or mineral structures [6]. Similarly, in pure cultures, the mycelium of *T. melanosporum* might use cellulose, cellobiose, lignin, chitin and tannins as carbon sources [7]. However, sequencing of the *T. melanosporum* genome showed that this fungus exhibits a restricted repertoire of genes coding for Carbohydrate Active enZymes (CAZymes) that are able to degrade dead organic matter [8]. *T. melanosporum* presents many fewer GH-encoding genes than saprotrophs, and cellulases from families GH6 and GH7 are absent [8]. These findings suggest that the saprotrophic ability of *T. melanosporum* is weak. In addition, *T. melanosprum* has an invertase gene that allows plant-derived sucrose to be hydrolyzed, suggesting the ability to use simple sugars from the host.

Our previous results based on the natural abundance of ^13^C and ^15^N in the ascocarp indicate that *T. melanosporum* behaves like a true ectomycorrhizal fungus and that the ascocarp cannot be mainly supplied *via* saprotrophic pathways from surrounding soil organic matter or dead host tissues. Our previous findings also suggest that *T. melanosporum* ascocarps cannot be completely independent at any time during their development, even during late maturation [9]. Similarly, *Tuber* ascocarps never develop when separated from their host [10].

However, *in situ* ^13^C and ^15^N labeling experiments are the only way to definitively answer the questions regarding carbon and nitrogen allocation during *Tuber* ascocarp differentiation. The technical difficulties inherent in this methodology are numerous, which is likely why no convincing experiments have yet been conducted to address this topic.

Numerous *in situ* ^13^CO_2_ pulse-labeling experiments have been conducted on annual crops or grasslands. These studies all demonstrated a rapid carbon flux pathway from the host to the roots [11], [12] and from the roots to the rhizosphere [12], [13]. Some of these studies include arbuscular mycorrhizas (AM). For example, Johnson et al. [14] showed that between 5 and 8% of the carbon lost by plants was respired by the AM mycelium over the first 21 h after labeling.

Several studies on carbon allocation in trees have been performed using pulse labeling. Most of these analyses were conducted in microcosms or mesocosms under controlled conditions with young seedlings. ^14^C pulse labeling followed by autoradiography or counting by scintillation was employed in these experiments [12], [15]–[24]. To our knowledge, very few studies have been conducted *in situ* with adult trees [25]–[28]. None of these experiments have considered the fructification of the associated fungi.

The aim of our work was to assess the allocation of carbon by the host to *T. melanosporum* mycorrhizas and ascocarps. This assessment was achieved *via* an *in situ* ^13^CO_2_ pulse-labeling experiment performed on a 20-year-old hazel tree in a truffle orchard established in the northeast of France.

## Materials and Methods

### The Experimental Site

The experiment was performed in Rollainville, which is situated in the west part of the Vosges in France on a limestone plateau of the Jurassic period (latitude 48° 18′ 42″; longitude 5° 44′ 13″; elevation 360 m; annual rainfall 941 mm with a maximum in July; mean annual temperature 9.5°C). The soil is a brown calcisoil (WRB 2006) with a silty clay texture, a high alkaline pH (water pH 7.97), a moderate content of organic matter (9.4%) and a limestone content of 8.8%. This soil is poor in available phosphorus and moderate in available potassium and magnesium. It is free-draining, highly granular and aerated.

The truffle orchard in which the experiment was conducted was established in 1991 by one of us (Christophe Robin). It was previously a cultivated site. No protected species were sampled. Hazel trees inoculated with *T. melanosporum* (Vittad.) (black Perigord truffle) marketed by the Naudet nurseries (http://www.pepinieres-naudet.com/) were planted [29]. The first truffle harvest began in November 2005.

### Labeling

In 2010, a single tree (A11, 4 m in height) was selected based on its high truffle producing. Two stainless steel scaffolds 6 m in height were built in parallel with one another on both sides (east and west) of the tree to install the labeling chamber. The two scaffolds were secured and attached to one another with stainless steel bars. The base of the chamber was sealed around the stems of the tree using adhesive tape with a width of 100 mm. The entire tree was enclosed in a 28-m^3^ cylindrical 200-µm polyethane film chamber into which pure ^13^CO_2_ gas was injected. The hazel tree was pulse-labeled first on the 10^th^ of July 2010 and a second time on the 1^st^ of September 2010. In July, the tree was watered one day before labeling (30 mm of water under the crown). The air temperature and air humidity inside the chamber were recorded with a single probe (HMP50, Vaisala, Finland) and a datalogger (CR1000, Campbell UK) at 30 s intervals. The labeling chamber was closed at 6∶36 UT for the first labeling and at 10∶41 UT for the second. Prior to injection, the CO_2_ concentration in the chamber was impoverished through leaf assimilation. Then, 15 l of ^13^CO_2_ (99 atom %, CORTECNET, France) were injected at a flow rate setting between 0.11 and 0.18 l min^−1^. Injection was initiated when the CO_2_ concentration reached 139 µmol.mol^−1^ for the first labeling (7∶40 UT) and 150 µmol mol^−1^ for the second (10∶57 UT). Total CO_2_ was regulated at 380 vpm using a ^13^CO_2_/^12^CO_2_ IRGA (S710, SICK/MAIHAC, Germany), and the evolution of the ^12^CO_2_ and ^13^CO_2_ concentrations was recorded inside the chamber. The concentration of ^13^CO_2_ inside the chamber reached 300 µmol mol^−1^ in the two labelings. Then, it declined to 73 µmol mol^−1^ during the first labeling event and 63 µmol mol^−1^ during the second. Finally, the chamber was opened at 9∶15 UT for the first labeling and 13∶04 UT for the second. The two labeling periods lasted 01∶45 h and 02∶07 h, respectively.

Based on the obtained data, the tree assimilated a total of 16.7 g of ^13^C during the two pulse-labeling periods. From October 2010 to March 2011, the crown of the tree was enclosed in a net to prevent any direct transfer of carbon to the soil through the falling of dead leaves, branches, nuts or catkins. These materials were collected regularly from the inside of the net. From December 2010 to February 2011, the soil under the tree was protected from frost using straw mulch (15 cm of straw enclosed in a plastic net).

### Sampling

Four quadrats of 1 m^2^ were positioned under the tree at the four cardinal directions (south, north, west and east) at a distance of one meter from the trunk. On eight dates (1, 4, 83, 101, 133, 168, 204 and 264 days from the first sampling performed on the 7^th^ of July 2010), ascocarps, ectomycorrhizal root tips, fine roots, bulk soil, mycorrhizospheric soil and ascocarpic soil were collected from the 0 to 10 cm depth in each of the four squares. During the same period, ascocarps were collected under control trees. Leaves, branches, catkins and buds were also collected in different periods and at the four positions (south, north, west and east).

#### Tree fine roots and mycorrhizas

Tree fine roots (≤2 mm diameter) and mycorrhizas were carefully retrieved from the soil and washed in water under a dissecting microscope. *Tuber melanosporum* mycorrhizas were identified *via* morphotyping on the basis of color, mantle shape and surface texture and some also by molecular typing. Fine roots and mycorrhizas were then treated for ten minutes with 1 M hydrochloric acid and then washed with water to eliminate soil calcium carbonate.

Mycorrhizas were confirmed as being associated with *T. melanosporum* using molecular methods. Genomic DNA was extracted with the DNeasy Mini Kit (Qiagen SA, Courtaboeuf, France) following the manufacturer’s instructions. *T. melanosporum* mycorrhizas were checked using species-specific ribosomal-DNA, internally transcribed-spacer (ITS) primers [30], [31]. A microsatellite genotyping of *T. melanosporum* mycorrhizas was performed using primer pairs corresponding to ten SSR markers. This data were then used to analyze the fine scale spatial genetic structure of *T. melanosporum* at the Rollainville site [32].

#### Soil

Bulk soil was collected at least 10 cm from any mycorrhizas or ascocarps. Mycorrhizospheric soil was obtained by carefully shaking roots with mycorrhizas and using needles or forceps. Ascocarpic soil was obtained by removing the soil adhering to the ascocarps using needles and forceps.

The soil samples collected in the field were immediately placed in an icebox and transferred to the laboratory at 4°C. After separation of the ascocarpic and mycorhizospheric soil, all of the samples were maintained at –80°C. They were not treated with hydrochloric acid.

#### Soil water extracts

To perform ^13^C and ^12^C measurements in soil water extracts, we used a portion of the samples held at –80°C. All of the samples were cleaned by removing small stones and shells using forceps under a dissecting microscope but were not treated by hydrochloric acid or ground. Living and dead mycorrhizas were removed from the mycorrhizospheric soil using forceps under a dissecting microscope.

For each sample, approximately 100 mg of soil was introduced into an Eppendorf tube with 0.5 ml of distilled water. The samples were shaken at 4°C for 24 h and then centrifuged for 5 minutes at 10,000 g. A 400 µl aliquot of the obtained supernatant was removed and immediately stored at −80°C. The 400 µl sample was then reduced to 100 µl using a cryodessicator and dried in a metal capsule. Each metal capsule was weighed before use and after drying to obtain the dry weight of organic matter dissolved in 400 µl. The dry weight of the soil introduced into each Eppendorf was also determined.

#### Ascocarps

During the 2010–2011 period of truffle production, 24 ascocarps produced beneath the labeled tree were found by chance inside the four squares or located by a dog outside of the four squares when mature. The ascocarps were carefully retrieved from the soil using a small garden trowel, as in the four squares. Harvesting was performed at five different times: 83, 101, 133, 168 and 204 days after the first labeling. During the following period of production (2011–2012), 3 ascocarps produced beneath the labeled tree were harvested (558 days after the first labeling). Ascocarps were also harvested under non labeled trees at three different times during the 2010–2011 period of production and at one time (01 16 2012) during following period of production. The ascocarps were also confirmed as belonging to T. melanosporum using molecular methods. The fresh weight of all of the ascocarps was determined after cleaning. Ten ascocarps were oven dried to obtain the average dry weight percentage (58.9%).

All of the ascocarps were also described morphologically and microscopically and classified using the following criteria (Table 1):

**Table 1:** Description of the maturation stages of *T. melanosporum* ascocarps (modified from Giovanni Pacioni, personal communication).

Stage	Description	Size or Weight
5a (Ascospores beginning to form)	Fertile veins with asci and ascospores beginning to form.	1 cm
5b (Smooth ascospores)	No echinulated ascospores; Brown-black peridium; White gleba; No aroma.	1–2 g
6a (Ornamented ascospores)	White echinulated ascospores; Brown-to-black peridium. Numerous open cracks. New wartsin formation; White-to-clear brown gleba; white veins clearly visible; Weak aroma.	2–5 g
6b (Ornamented brown ascospores)	Black-brown peridium with few closed cracks; Grey-black gleba between white veins;80% echinulated ascopsores, brown to dark brown; 20% of white echinulated ascopsores;Fairly developed aroma; Not completely mature.	5–15 g
6c (Brown-to-dark brown echinulated ascospores)	Black-brown peridium with few closed cracks; Black gleba between white veins; Very welldeveloped aroma; Completely mature.	15–100 g and more

The first stages (truffles of less than 1 g) could not be harvested. We attempted to quantify the constitutive carbon derived by the ascocarps from the host by assuming that, during the 2010–2011 period of production, the 24 ascocarps were present at the time of the first crop and that they all grew in a synchronized manner. The ascocarps of the last crop were partly desynchronized from the previous ascocarps. Their growth was slowed by low temperatures. Consequently, we excluded the last harvest and considered only the 18 ascocarps harvested from 28 September to 22 December 2010. We also considered only the gleba in the calculations, as the weight of the peridium was negligible. We used the average C concentration in the 18 ascocarps cropped under the labeled tree (43.48% C). For each date, the accumulated weight of constitutive carbon in the ascocarps (ΣCW) was as follows:
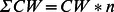
where CW is the average constitutive carbon on that date, and n is the total number of ascocarps harvested from the beginning of the study.

On each date, the weight of ^13^C derived from the host (^13^CW) was calculated as follows:
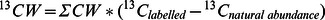
where Σ*CW* is the accumulated weight of constitutive carbon in the ascocarps; ^13^C_labelled_ is the measured ^13^C abundance on each date; and ^13^C_natural abundance_ is the natural abundance.

#### Leaves

On each date, ten leaves were collected around the crown in the middle part of the tree at the four cardinal points and pooled together to obtain one sample per cardinal point.

For each date, there were four replicates of each type of material (leaves, fine roots, mycorrhizas, soil, soil solutions), with the exception of ascocarps, the number of which depended on the harvest. The samples were first air dried, then dried at 60°C for 48 h and ground to a fine powder using a shaker with steel beads.

#### Isotopic analysis

The percentages of total C and the C isotopic compositions in the leaves, fine roots, mycorrhizas, ascocarps, bulk soil, mycorrhizospheric soil, ascocarpic soil and solutions of bulk, myco-rhizospheric and ascocarpic soil were determined at INRA Nancy using an online continuous flow CN analyzer (Carlo Erba NA 1500) coupled to an isotope ratio mass spectrometer (Finnigan delta S). Values were reported using standard notation (δ^13^C ‰) relative to Vienna PeeDee Belemnite (VPDB), employing polyethylene foil (IAEA-CH-7) as a standard.

δ^13^C values were calculated with the usual formula:
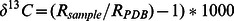
where R is the molar ratio of ^13^C/^12^C, and R_PDB_ is the molar ratio of PeeDee Belemnite. For ascocarps, the natural abundance δ^13^C (‰) value was calculated by averaging the δ^13^C values of ten ascocarps collected during the same period beneath an unlabeled hazel tree. For each organ, Excess (‰) δ^13^C = δ^13^C_labeled_ –δ^13^C_natural abundance_. For soil samples, the amount of soluble ^13^C was expressed in nanograms per 100 mg of dried soil.

### Statistical Analyses

Analyses of variance for experimental data were conducted using the R software (R project for Statistical computing, http://www.R-project.org). Analyses of variance were performed using Type-II sum of squares (Anova function from package "car") when data were missing, causing unbalanced design. When necessary, data were transformed prior to the Anova using the Box-Cox method [33]. The criterion for statistical significance was set at p<0.05.

## Results

The flux of pulse-derived ^13^C from the leaves to the fine roots, mycorrhizas, ascocarps and soil was traced and quantified over a seven-month post-labeling period.

### Leaves

The natural δ^13^C abundance in the leaves presented an average value of −27.66 ‰ prior to the first labeling (Table 2). Leaf δ^13^C reached a level of almost 300 ‰ just after the end of the first ^13^CO_2_ injection, after which it decreased rapidly (to 35 ‰ after 5 days) but remained positive until the second labeling, when it peaked at 470 ‰. The δ^13^C subsequently decreased until reaching a negative value at leaf fall. Dormant buds sampled during winter showed high ^13^C abundance, as did branches formed in 2010 whose ^13^C concentration was higher than in older branches. The δ^13^C reached 76 ‰ in the following spring in the newly formed leaves, just after bud break.

**Table 2:** Kinetics of δ^13^C (in ‰) in leaves of the hazel tree A11 after pulse labeling and δ^13^C of buds and branches sampled during the winter following the pulses.

Time in days from thefirst labeling	−3	0	5	18	52	80	130	210	261
Time in days from thesecond labeling					0	28	78	158	209
Leaves (‰)	−27.66(1.51) a	290.71(173.00) b	35.26(55.63) a	29.65(57.88) a	469.07(211.2) b	11.45(12.75) a	−4.45(12.45) a		76.70(43.24) a
Dormant buds (‰)								45.53 (18.36)	
2010 branches (‰)								13.81 (10.88)	
2009 branches (‰)								−9.11 (4.88)	
2008 branches (‰)								11.96 (5.37)	
2005 branches (‰)								16.84 (2.66)	

One-way Anova has been performed using R; data have been raised to power minus 2 prior to Anova as suggested by the Box-Cox method in order to ensure the normality of residuals. Standard errors of means are given in brackets and mean values followed by a different letter are significantly different from the others at p<0.05 (Mean comparison Tukey’s test).

### Fine Roots and Mycorrhizas

Mycorrhizas were significantly more labeled than fine roots (p<0.01) and there was a date effect (p<0.001) but no interaction (Table 3). A nonsignificant increase of the δ^13^C was visible in fine roots sampled 26 days after the first labeling. The δ^13^C level in fine roots always remained below zero throughout the period following the first ^13^C pulse. The δ^13^C was higher after the second labeling, from the sampling at 149 days after the second pulse where it peaked at 9.87 ‰ (January 2011). There was a transfer of ^13^C to the *T. melanosporum* mycorrhizas that became positive 80 days and 165 days after the first labeling (113 days after the second one). The mycorrhizal δ^13^C level peaked at +22.75 ‰, 80 days after the first pulse and then decreased. It increased again after the second labeling, reaching a maximum of +55.35 ‰ prior to decreasing again.

**Table 3:** Kinetics of δ^13^C (in ‰) in the fine roots and *T. melanosporum* mycorrhizas beneath hazel tree A11 in 2010–2011 after the pulse labelings of the leaves with ^13^CO_2_.

Time in days from the first labeling	−3	1	26	80	130	165	201	261
Time in days from the second labeling				28	78	113	149	209
Fine roots (‰)	−27.62(0.29) a	−26.62(2.28) a	− 9.73(33.52) ab	−13.15(13.60) ab	−19.34(3.51) ab	− 10.12(12.06) ab	9.87(41.20) ab	3.82(17.52) b
Mycorrhizas (‰)	−27.60(0.19) a	−24.09(5.35) a	−24.60(2.24) a	22.75(47.97) b	−5.31(19.33) b	26.3(12.08) b	52.35(35.06) b	18.85(7.23) b

Two-way Anova has been performed using R; data have been raised to the power minus 2 prior to Anova as suggested by the Box-Cox method in order to ensure the normality of residuals. Anova showed a ‘date’ (p<0.01) and ‘organ’ (p<0.001) effects but no interaction. Standard errors of means are given (in brackets). Mean comparison has been made for simple effects (Tukey test): for d^13^C in fine roots and d^13^C in mycorrhizas respectively, means followed by a different letter are significantly different.

### Ascocarps

The first ascocarps beneath the labeled tree were harvested on the 28^th^ of September 2010 and the last on the 27^th^ of January, 2011 (Table 4). The first ascocarps were immature (stages 5b to 6a). They matured gradually, and in January 2011, all of the harvested ascocarps were fully ripened (stage 6c). The synchronization between the ascocarps was not complete, and there were some variations in the maturation stage on each date.

**Table 4:** (A) Maturity, numbers and fresh weight of ascocarps harvested beneath the labeled tree A11; (B) δ^13^C (in ‰) in *T. melanosporum* ascocarps (peridium and gleba) beneath the labeled tree A11, and beneath non-labeled trees (natural abundance) at each sampling date from October 2010 to January 2011.

		2010				2011	2012
	Harvest date	Sept-28	Oct-16	Nov-17	Dec-22	Jan-27	Jan-16
**A**	Time in days from the first labeling	80	98	130	165	201	555
	Time in days from the second labeling	28	46	78	113	149	503
	Stage of maturity	5b to 6a	6a	6a to 6b	6b to 6c	6c	
	Average fresh weight (g)	1.95	10.6	15.3	35.5	19.4	
	Number of ascocarps harvested	4	5	3	6	6	
	Fresh weight harvested (g)	7.8	63	45.9	213	116	
	Accumulated fresh weight harvested (g)	7.8	70.8	116.7	330	446	
**B**	δ^13^C in the peridium in ‰, labeled tree A11	+87.01 (34.23) b	+60.36 (12.67) b	+69.56 (34.30) b	+59.43 (6.35) b	+77.36 (26.08) b	−25.84 (0.37) b
	δ^13^C in the gleba in ‰, labeled tree A11	+125.4 (29.93) c	+78.94 (12.33) b	+79.13 (22.37) b	+67.17 (15.67) b	+82.12 (27.87) b	−26.22 (0.46) a
							
	δ^13^C in the peridium in ‰, Controls		−26.11 (0.32)		−25.65 (0.60)	−25.84 (0.87)	−26.16 (0.61)
	δ^13^C in the gleba in ‰, Controls		−25.71 (0.18)		−24.93 (0.70)	−25.39 (0.34)	−25.79 (0.83)

n = 3 to 7 ascocarps harvested at each sampling date. Two-way Anova has been performed using R (table 4B); data have been log transformed prior to Anova as suggested by the Box-Cox method in order to ensure the normality of residuals. Anova showed a ‘date’ (p<0.05) and ‘organ’ (p<0.001) effects but no interaction. Standard errors of means are given (in brackets). Mean comparison has been made for simple effects (Tukey test): for δ^13^C in the peridium and δ^13^C in the gleba, means followed by a different letter are significantly different.

At the end of September, the average fresh weight of the ascocarps was less than two grams. The ascocarps continued to grow until the end of December to reach an average of 35 g. The ascocarps harvested at the end of January 2011 were smaller than those from December 2010, most likely due to low soil temperatures, differences in primordium production times and maturation desynchronization among the different ascocarps. The 24 collected ascocarps produced a total of 446 g fresh weight.

In the peridium and the gleba, the δ^13^C was highest in the first harvest (87 and 125‰ respectively). It subsequently decreased and then increased again after the second labeling. The ^13^C enrichment was significantly higher in the gleba than in the peridium (p = 0.034), and the δ^13^C in the peridium and gleba were significantly higher than natural abundance, whatever the sampling date.

At the end of December, the gleba of the ascocarps, which had reached full growth, were 35% more enriched in ^13^C than the mycorrhizas and almost three times more enriched than the fine roots. The δ^13^C in the ascocarps harvested in 2012 under the labeled tree, 555 days after the first labeling, displayed a value identical to the natural abundance. In the controls, the δ^13^C values in the ascocarps were not different between the gleba and the peridium and remained very stable between −25 and −26‰ throughout the period of maturation.

### Constitutive ^13^C Derived by the Ascocarps from the Host

From the 28^th^ of October to the 22^th^ of December, 2010, the ascocarps continuously absorbed ^13^C from the host (Table 5). A total of 160.4 mg of ^13^C was derived to provide constitutive carbon to the ascocarps at a flux of 1.7 mg per day until the 22^nd^ of December, representing 0.96% of the ^13^C assimilated by the tree during the two labeling periods. The ^13^C allocated to the ascocarps to provide constitutive carbon was even higher, as there were only 18 fruiting bodies considered in this case, instead of 24. The increase in derived ^13^C used as constitutive carbon in the ascocarps was similar to the increase in total constitutive carbon.

**Table 5:** Characteristics of the *T. melanosporum* ascocarps harvested in 2010–2011 beneath the labeled hazel tree A11 and estimations of the amounts of ascocarpic ^13^C derived from the host tree.

	2010				2011
Harvest date	Sept-28	Oct-16	Nov-17	Dec-22	Jan-27
Number of ascocarps harvested	4	5	3	6	6
Average dry weight (g)	1.15	6.24	9.00	30.10	11.42
Ascocarp dry weight harvested (g; 24 ascocarps)	4.59	37.08	27.02	73.82	68.73
Accumulated ascocarp dry weight (g; 24 ascocarps)	4.59	41.67	68.69	183.97	252.70
Accumulated dry weight in g (18 ascocarps)	20.70	112.32	162.00	361.80	
Accumulated constitutive carbon in the ascocarps in g (SCW)(18 ascocarps)	9.00	48.84	70.44	157.38	
^13^C derived from the host in mg (^13^CW)(18 ascocarps)	14.81	55.59	82.66	160.37	

### From Fine Roots to Ascocarps

**Figure 1:**
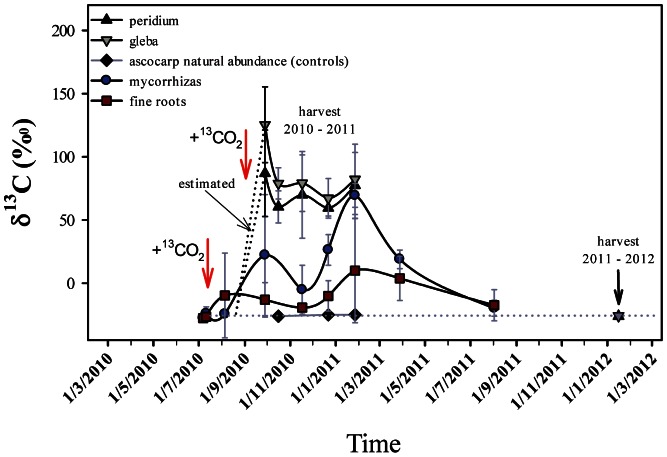
Temporal variation of δ^13^C (‰) in the fine roots, mycorrhizas and peridium and gleba of ascocarps beneath hazel tree A11 in 2010–2011. Error bars represent the standard deviation of the means.

Despite appearing nonstatistically significant, the transfer of^ 13^C was effective in the fine roots, which never accumulated ^13^C (Figure 1). The transfer of ^13^C from the fine roots to the mycorrhizas was delayed for several weeks. From the mycorrhizas to the ascocarps, the ^13^C transfer was intensive 80 days after the first injection of ^13^CO_2_. It then decreased, followed by increasing from days 113 to 149 after the second labeling. It was not possible to harvest truffles in early stages (less than 1 g). The early transfer of ^13^C from the host towards the ascocarps probably occurred at least 60 days after the first labeling.

### Soil and Soil Water Extracts

The δ^13^C values did not differ significantly between the three soil compartments (bulk, mycorhizospheric and ascocarpic soil) and remained constant (average −25.22‰) from July 7 2010 (before the first labeling) to January 2011 (Table 6). There was a date effect (p = 0.0011), the last sampling date presenting higher δ^13^C values than at 1, 98 and 130 days after the first labeling.

**Table 6:** δ^13^C (‰) in the soil compartments (bulk soil, mycorhizospheric soil, soil adhering to the ascocarps) following the pulse labelings of the A11 hazel tree.

	2010						2011	
Harvest date	July-07	July-10	Sept-28	Oct-16	Nov-17	Dec-22	Jan-27	March-28
Time in days from the first labeling	−3	1	80	98	130	165	201	261
Time in days from the second labeling			28	46	78	113	149	209
δ^13^C in the bulk soil (‰)	−24.46 (0.80)	−25.13 (0.80)	−25.71	−26.28 (1.26)	−25.98 (0.42)	−25.29 (0.89)	−25.15 (0.43)	−24.33 (0.39)
δ^13^C in the myco-rhizospheric soil (‰)	−25.53 (1.29)	−26.51 (0.38)	−26.96 (0.38)		−26.17 (0.48)	−23.74		−23.55 (1.99)
δ^13^C in the adherent ascocarpic soil (‰)				−25.84 (0.82)	−26.07 (0.73)	−24.39 (1.98)	−24.06 (3.14)	

Two-way Anova has been performed using R; data have been log transformed prior to Anova as suggested by the Box-Cox method in order to ensure the normality of residuals. Anova showed a ‘date’ effect (p = 0.0011) but no ‘organ effect’ and no interaction. Analysis of variance was performed using Type-II sum of squares (Anova function from package "car") because of unbalanced design resulting from missing data. When available, standard errors of means are given (in brackets).

The C content in water soil extracts was higher in the mycorhizospheric soil (173.5 ng per 100 mg of dry soil) than in the bulk soil (107.9 ng per 100 mg of dried soil) (p<0.001) or in the ascocarpic soil (131.7 ng per 100 mg of dried soil) (p<0.05).

The δ^13^C values in the soil water extracts did not differ significantly between the three compartments and remained constant throughout the period investigated, with an average value of −24.45‰ (Table 7).

**Table 7:** δ^13^C (‰) in soil water extracts following the pulse labeling of the A11 hazel tree.

	2010						2011	
Harvest date	July-07	July-10	Sept-28	Oct-16	Nov-17	Dec-22	Jan-27	March-28
Time in days from the first labeling	−3	1	80	98	130	165	201	261
Time in days from the second labeling			28	46	78	113	149	209
δ^13^C of the bulk soil solution (‰)	−25.20 (1.02)	−24.73 (1.73)	−24.97 (1.02)	−24.09 (1.94)	−24.89 0.79)	−24.68 (1.97)	−25.75 (0.71)	−25.25 (0.40)
Soluble ^13^C (ng for 100 mg soil)	90.5 (25.1)	83.6 (11.8)	105.2 (14.8)	159.5 (56.9)	86.1 (25.8)	69.2 (13.4)	83.7 (10.0)	185.9 (30.3)
δ^13^C of the myco-rhizospheric soil solution (‰)	−25.24 (0.95)	−25.24 (0.28)	−23.49 (0.66)		−25.00 (0.61)	−25.55		−22.76 (4.15)
Soluble ^13^C (ng for 100 mg soil)	177.5	134.2	253.0		168.6 (73.0)	103.6		221.1 (95.9)
δ^13^C of the ascocarpic soil solution (‰)				−24.40 (0.67)	−24.08 (0.18)	−22.13 (3.88)	−22.68 (3.77)	
Soluble ^13^C (ng for 100 mg soil)				141.9 (7.7)	112.6 (23.9)	146.6 (50.3)	125.9 (27.9)	

Two-way Anova has been performed using R; data have been log transformed prior to Anova as suggested by the Box-Cox method in order to ensure the normality of residuals. Anova showed no main effects and no interaction. Analysis of variance was performed using Type-II sum of squares (Anova function from package "car") because of unbalanced design resulting from missing data. Anova showed a slight date effect (p = 0.046) and no interaction. When available, standard errors of means are given (in brackets).

## Discussion and Conclusions

It is accepted that ectomycorrhizal fungi do not rely on dead organic matter as a carbon source. Using ^14^C as a tracer in forest conditions (Oak Ridge Reservation, Tennessee), Treseder et al. [34] demonstrated that basidiomycota ectomycorrhizal fungi acquired most or all of their carbon from their hosts and that less than 2% of the carbon in the ectomycorrhizal biomass originated from the litter. Similarly, in several studies examining changes in photoperiods or photosynthesis rates, the defoliation or girdling of the host suggested that basidiomycota sporocarps depend strongly on newly synthesized carbon from the host [35]–[39]. However, 75% defoliation of a *Pinus pinaster* stand affected the mycorrhizal community but did not decrease basidiomycota sporocarp biomass or abundance [40].

However, Hobbie et al. [41], using the ^14^C signal from 1950s to 1960s thermonuclear testing, suggested that some ectomycorrhizal fungi like *Cortinarius*, *Leccinum* or *Tuber* could be able to use some insoluble soil organic matter.

In our experiment, the transfer of ^13^C from the hazel tree leaves to the mycorrhizas via the fine roots was very slow. This result contrasts with what is observed in herbaceous plants, in which allocation to the roots is far more rapid [11], [13]. Furthermore, the carbon allocation by the host to the ectomycorrhizas appeared to take place very slowly compared to what has been found in arbuscular mycorrhizal plants. For example, Leake et al. [18] observed peak transfer from cores colonized by AM mycelium 9–14 h after labeling. This transfer was also slower than the transfer observed by Högberg et al. [25] in a *Pinus sylvestris* forest, where the ^13^C content peaked after 4–7 days in ectomycorrhizal pine roots.

In our experiment, the fine roots acted mainly as a conduit. They did not accumulate ^13^C and transferred it slowly to the mycorrhizas. The mycorrhizas first formed a carbon sink and accumulated ^13^C prior to ascocarp development. Then, the mycorrhizas transferred ^13^C to the truffles, which accumulated carbon from the host until reaching complete maturity, 200 days after the first labeling and 150 days after the second labeling. This role of the *Tuber* ascocarps as a carbon sink was observed several months after the end of carbon assimilation by the host, which lost its leaves, and at low temperature. This finding suggests that the carbon allocated to the ascocarps during fall and winter was provided by reserve compounds stored in the trunk, branches, buds or thick roots. In the labeled tree, there was ^13^C accumulation in the dormant buds and in the newly formed branches.

Tissues of deciduous trees store starch in autumn [42]. This starch is partly hydrolyzed during dormancy. In poplar trees during autumn and winter, starch hydrolysis results in a huge increase in sucrose and its galactosides [43]. This process represents a mechanism for protection against frost [44]. Starch resynthesis occurs at the end of dormancy, and new hydrolysis of starch into simple sugars is observed at bud break [45]–[47]. Carbon remobilization in deciduous trees could provide up to approximately 40% of the C used for new tissue formation and can contribute to early wood formation [48]–[50]. Under the conditions of the present study, we can assume that the carbon allocated by the host to *T. melanosporum* ascocarps at the end of their development is in the form of simple sugars produced *via* starch hydrolysis during tree dormancy, rather than from amylase activity prior to or during bud breaking. Nevertheless, under a Mediterranean climate, the carbon used for growth of *T. melanosporum* ascocarps could enter into competition with the carbon necessary for bud bursting or early wood formation. However, the quantity of carbon necessary for ascocarp development (approximately 1% of the assimilated C) is not comparable to the quantity of C necessary for bud breaking. In 2012, when the ascocarp δ^13^C content was found to be equal to the natural abundance, it became clear that the carbon allocated to the fruiting bodies was only coming from the carbon assimilated by the tree during the growing season.

Using *Pinus densiflora* seedlings in mycorrhizal association with *Laccaria amethystina* and labeled with ^14^CO_2_, Teramoto et al. [3] showed that there was transfer of host carbon to the sporocarps over one or a number of days in rhizoboxes based on autoradiography and radioactivity counting. This finding indicates that the fungus primarily used recently assimilated carbon. This result obtained in an ectomycorhizal member of Basidiomycotina producing fruitbodies over a number of days contrasts with our results obtained with an ectomycorrhizal member of the Ascomycotina, in which the development of ascocarps requires carbon stored in the trunk or roots of the host, and the process takes several weeks/months.

These results demonstrate, for the first time under field conditions, that *Tuber* mycorrhizas provide a slow, but dominant pathway for carbon flux from trees to ascocarps. From September 28 2010 until December 22 2010, 1.7 mg ^13^C was transferred per day from the mycorrhizas to the ascocarps to provide constitutive carbon. The total amount of constitutive ^13^C transferred was approximately 1% of the ^13^C assimilated by the tree during the two labeling periods. These data do not include the carbon respired by the ascocarps.

This ^13^CO_2_ pulse-labeling experiment corroborates our previous results based on natural ^13^C and ^15^N abundance showing that carbon allocation for ascocarp development could not be supplied mainly *via* saprotrophic pathways [9]. According to our pulse-labeling experiment, almost all of the carbon allocated to the truffle ascocarps came from the host. These findings also corroborate the results of sequencing of the *T. melanosporum* genome, which showed that this fungus has a limited repertoire of genes coding for CAZymes [8]. Nevertheless, we cannot exclude the possibility of weak carbon allocation to the ascocarps from soil organic matter. Soil labeling experiments (^15^N and ^13^C) are necessary to determine whether truffle ascocarps can also use dead host tissues or soil organic matter as carbon and nitrogen sources.

Several questions remain concerning the mode of carbon transfer between the host tree and developing ascocarps. Based on our results, it is clear that this transfer cannot occur through the soil. The soil and the soil solutions never appeared to be enriched in ^13^C, regardless of the compartment considered. Epron et al. [26] also observed an absence of bulk soil enrichment after ^13^CO_2_ pulse labeling of three species (*Fagus sylvatica*, *Quercus petraea* and *Pinus pinaster*).

This transfer also cannot take place *via* the external mycelium of the mycorrhizas. This mycelium extends to a maximum of a few millimeters from the mantle, and the ascocarps generally develop at least several cm from the mycorrhizas. The most likely hypothesis to explain this type of carbon transfer is a transport through the ascogonial structure, which could provide a direct connection between mycorrhizas and ascocarps.

In conclusion, it appears evident that *Tuber* ascocarps are dependent on their hosts throughout their development. These results contradict the statements of well-recognized truffle handbooks and could be of some importance for improvement of truffle cultivation methods, for example, through using caution regarding tree pruning at truffle primordium production and during truffle growth.
